# Higher mortality rates among the elderly with mild traumatic brain injury: a nationwide cohort study

**DOI:** 10.1186/1757-7241-22-7

**Published:** 2014-01-28

**Authors:** Po-Liang Cheng, Hsin-Yi Lin, Yi-Kung Lee, Chen-Yang Hsu, Ching-Chih Lee, Yung-Cheng Su

**Affiliations:** 1 Emergency Department, Dalin Tzu Chi Hospital, Buddhist Tzu Chi Medical Foundation, No.2, Minsheng Rd, Dalin Township, Chiayi County 622, Taiwan; 2 School of Medicine, Tzu Chi University, Hualien, Taiwan; 3 Department of Public Heath, National Taiwan University, Taipei, Taiwan; 4 Community Medicine Research Center and Institute of Public Health, National Yang-Ming University, Taipei, Taiwan; 5 Department of Otolaryngology, Dalin Tzu Chi Hospital, Buddhist Tzu Chi Medical Foundation, Chiayi, Taiwan; 6 Cancer Center, Dalin Tzu Chi Hospital, Buddhist Tzu Chi Medical Foundation, Chiayi, Taiwan

**Keywords:** Mortality, Traumatic brain injury

## Abstract

**Background:**

It is known that the risk of death in elderly patients with moderate to severe traumatic brain injury is increased. However, the relationship between mild traumatic brain injury and death has never been established. We investigated the mortality rates of older patients with mild traumatic brain injury in Taiwan to evaluate if there is a higher risk of death compared with the general population.

**Methods:**

We utilized a sampled National Health Insurance claims database containing one million beneficiaries. We followed all adult beneficiaries older than 65 years from January 1, 2005 till December 31, 2009 to see if they died. We further identified patients with mild traumatic brain injury and compared their risk of death with the general population.

**Results:**

We identified 5997 patients with mild traumatic brain injury and 84,117 patients without mild traumatic brain injury. After controlling for age, gender, urbanization level, socioeconomic status, diabetes, hypertension, history of alcohol intoxication, history of ischemic stroke, history of intracranial hemorrhage, malignancies, dementia and Charlson Comorbidity Index score, the adjusted hazard ratio was 1.25 (95% confidence interval, 1.16—1.34).

**Conclusions:**

Mild traumatic brain injury is an independent significant risk factor for death in the elderly.

## Introduction

Each year, traumatic brain injury (TBI) accounts for 2.4 million emergency department visits, hospitalizations, or deaths in the United States, and the direct medical costs of TBI in 2010 were estimated to be $11.5 billion [1]. With growing aging populations, especially in the developed countries, understanding the impacts of TBI in the elderly and efforts to reduce the rates of TBI are both important. In one study, patients older than 65 years with severe TBI had a 72% greater chance of death compared with younger patients [2]. In other studies, age at the time of injury was also found to be associated with a higher risk of death after TBI, which shows that older patients are more vulnerable to TBI [3-5].

Although the association of TBI with risk of death has been noted, it may be argued that the effect in older patients could be partly explained by pre-existing co-morbidities [6-8]. In a study by Selassie et al., [3] specific chronic diseases were associated with postdischarge mortality after TBI. Furthermore, the relationship of mild TBI (the majority of TBI cases) to death is inconclusive. In a population-based controlled study, the hazard ratio (HR) of death was statistically significant in patients with moderate to severe TBI but not in patients with mild TBI. However, the finding may be limited since the number of deaths in the study was small [9].

Our study aimed to investigate the correlation between mild TBI and death in the elderly by utilizing a large administration database to overcome the obstacles noted above. Adjusted HRs were used to compare the risk of death between older patients with mild TBI and a control group over a five-year period. The results of this study might provide clinicians with further insights into this frequently encountered situation.

## Methods

### Ethics statement

This study was initiated after approval from the Institutional Review Board of Buddhist Dalin Tzu Chi General Hospital, Taiwan. Since all personal identification was stripped from the secondary files before analysis, the review board waived the requirement of written informed consent from the patients.

### Database

The National Health Insurance (NHI) program was implemented in Taiwan in 1995 and provides compulsory universal health insurance. It enrolls up to 99% of the Taiwanese population and contracts with 97% of all medical providers [10,11]. The database contains comprehensive information on all insured subjects, including sex, date of birth, residential or work location, dates of clinical visits, the International Classification of Diseases (Ninth Revision) Clinical Modification (ICD-9-CM) diagnostic codes, details of prescribed medications, expenditure amounts and outcome at hospital discharge (i.e., recovered, died, or transferred out). A random sample consisting of one million people based on the 2005 reimbursement data was established for public access; the group did not significantly differ statistically from the larger cohort in age, gender or health care costs according to the Taiwan National Health Research Institute. A sampled group was used as our study cohort.

### Study population

**Figure 1:**
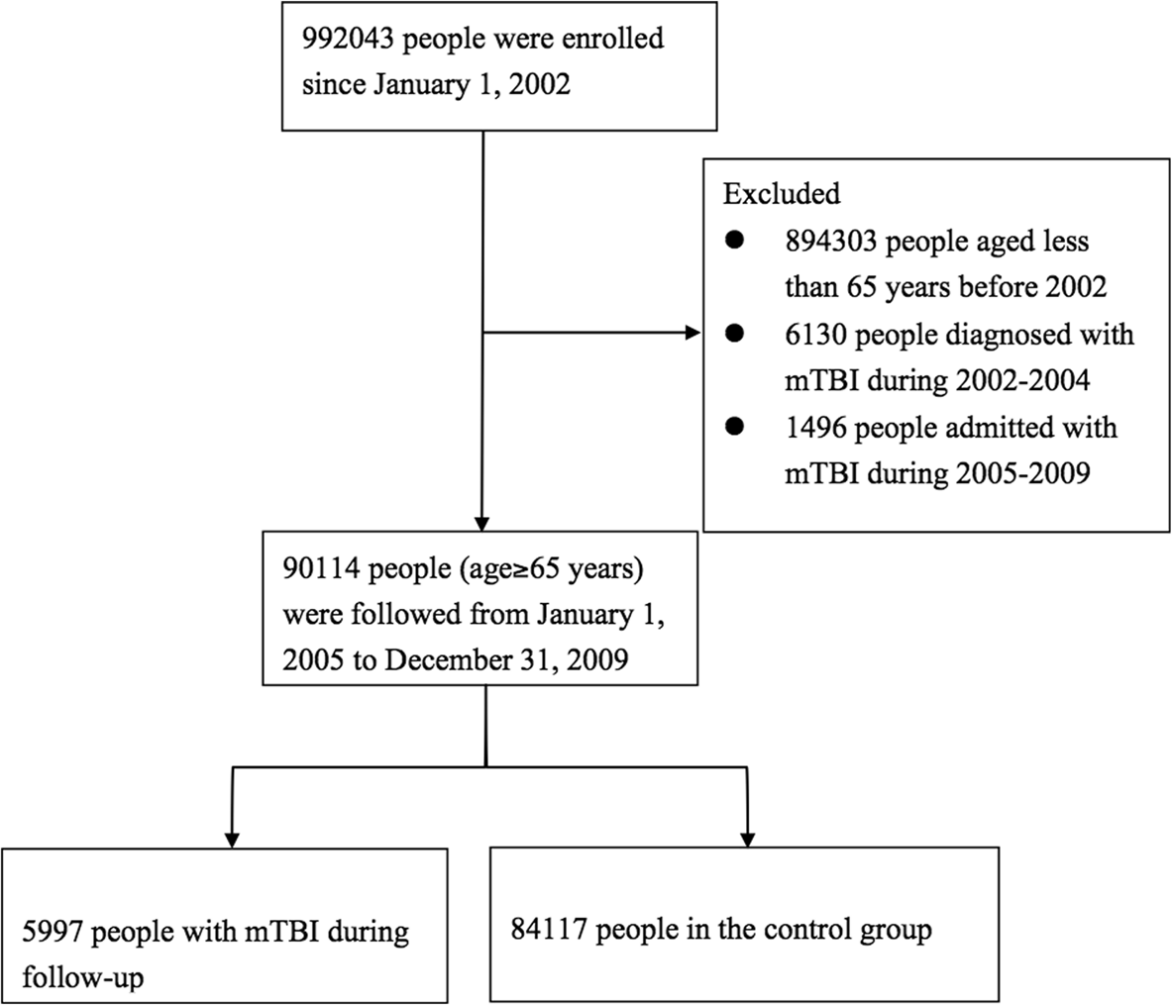
Flow diagram of population-based study.

The sampled population was followed from January 1, 2002 to December 31, 2009 (a total of 8 years). First, we identified individuals for our study cohort who were still alive in 2005 and were aged older than 65 years. Mild TBI was defined by ICD-9-CM code head concussion (850.0, 850.1, 850.5, or 850.9), intracranial injury of other and unspecified nature (854.0), or head injury, unspecified (959.01) [12,13]. We excluded patients with mild TBI diagnosed before January 1, 2005. In order to avoid misclassification, we also excluded patients who had ever been hospitalized with TBI to ensure that enrolled patients with mild TBI were discharged directly after visits. After exclusion of our cohort cases, we identified 5997 patients with mild TBI and 84,117 without mild TBI. Each was tracked from the date of mild TBI or January 1, 2005 (baseline) until December 31, 2009 (study end) to determine if the patient had died during this period. Cases were censored for patients who either drew back guarantees from the NHI Program or were still robust at end of the follow-up period (Figure 1).

### Covariates

To better understand the effect of mild TBI on the risk of death, this study used several covariates. These included patient demographics such as age, sex, urbanization level (i.e., urban, suburban, and rural areas) and socioeconomic status (SES). This study used the income-related insurance payment amounts as a proxy measure of individual SES at follow-up. People were classified into three groups: (1) low SES: payment lower than US$571 per month (New Taiwan Dollars [NT$] 20,000); (2) moderate SES: payment between US$571–1141 per month (NT$ 20,000–40,000); and (3) high SES: US$1142 or more payment per month (NT$40,001) or more [14]. Second, the prevalence of selected comorbid conditions (i.e., diabetes, hypertension, history of alcohol intoxication, history of ischemic stroke, history of intracranial hemorrhage, malignancies and dementia) and the Charlson Comorbidity Index (CCI) score were determined according to the discharge diagnosis either during outpatient clinic visits or hospitalizations before January 1, 2005. The CCI is a scoring system that includes weighing factors on important concomitant diseases; it has been validated for use with ICD-9-CM coded administrative databases [15,16].

### Statistical analysis

The SAS statistical package, version 9.2 (SAS Institute, Inc., Cary, NC, USA), and STATA version 11.2 (StataCorp, College Station, TX, USA) were both used for data analysis. All covariates were taken as categorical variables except age, which was treated as a continuous variable. Categorical variables were compared with Pearson’s chi-square test and continuous variables with the t test to reveal the baseline heterogeneity in the two groups. Kaplan-Meier curves were first plotted to show the trend of death. Cox proportional hazard regression models were then used to calculate the HRs for people with mild TBI after adjustments for age, gender, urbanization level, SES, diabetes, hypertension, history of alcohol intoxication, history of ischemic stroke, history of intracranial hemorrhage, malignancies, dementia and CCI. Adjusted HRs were analyzed both for 1) from mild TBI or baseline through study end, and 2) from 6 months after mild TBI or baseline through study end.

To further assess the robustness of our results, we performed a subgroup analysis to evaluate the risk of death in patients who had been hospitalized because of TBI to see if there is a ‘dose-response’ effect in the relationship between TBI and death. We also performed sensitivity analyses [17,18] to evaluate how large the effect of an unmeasured confounder would be to account for the results. A two-tailed P value of <0.05 was considered significant.

## Results

**Figure 2:**
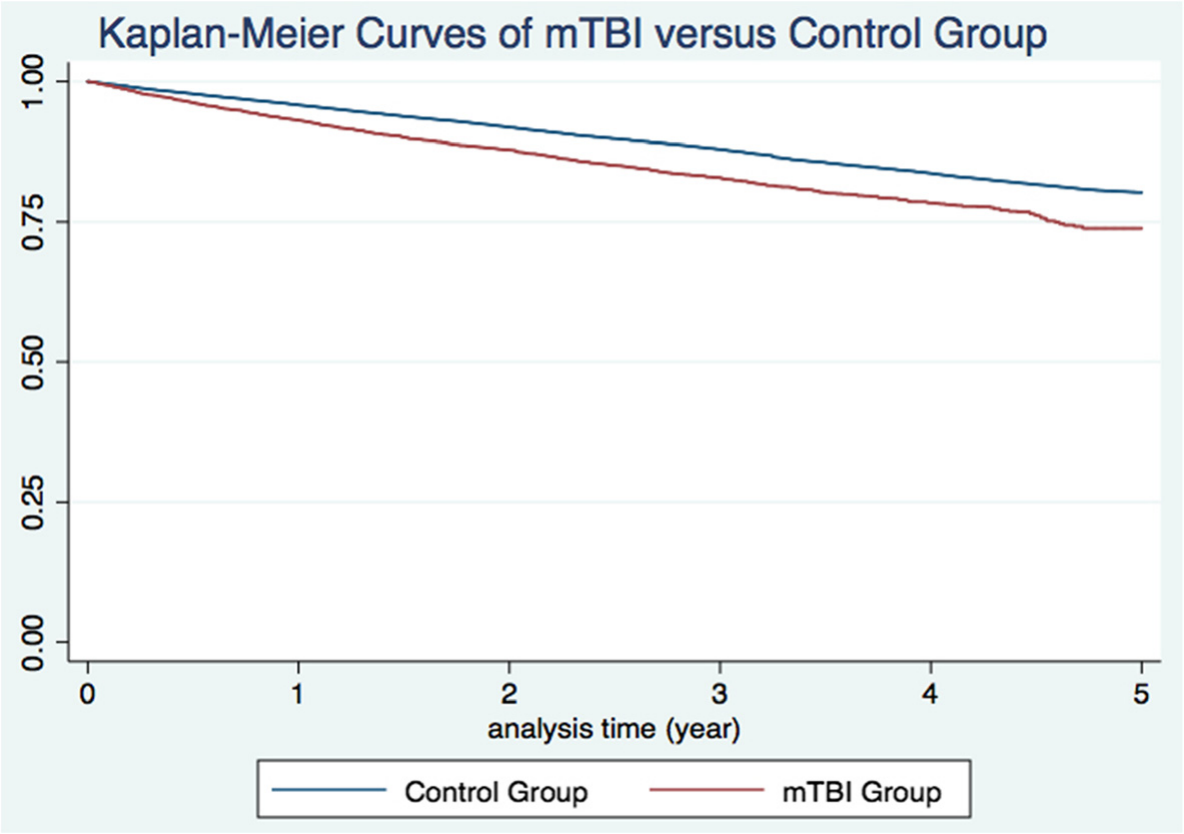
Survival curves of mild TBI group and control group.

The distribution of both demographic characteristics and selected morbidities is shown in Table 1. There were 5997 patients in the mild TBI group and 84,117 in the control group. Total follow-up periods in the two groups were 12,989 and 377,279 person-years, respectively. The percentage of patients who underwent computed tomography (CT) examinations in the mild TBI group was 13.7%. Patients with mild TBI were significantly older and also significantly more likely to have diabetes, hypertension, history of alcohol intoxication, ischemic stroke, intracranial hemorrhage, dementia, and higher CCI score. By the end of follow-up, 17,465 patients had died, including 868 in the mild TBI group and 16,597 in the control group. The average duration from mild TBI to death was 1.40 years (95% confidence interval [CI], 1.32—1.48). The crude HR of death between the two groups was 1.51 (95% CI, 1.42—1.62). Kaplan-Meier curves showed a higher trend of death in the mild TBI group (Figure 2).

**Table 1:** Baseline characteristics of the mild TBI group and control group

**Variables**	**Mild TBI group (n =5997)**		**Control group (n = 84117)**		***P*-value**
	**No.**	**%**	**No.**	**%**	
Male	2851	47.5	41988	49.9	<0.001
Mean age (SD)	75.8	7.3	73.6	6.5	<0.001
Socioeconomic status					<0.001
Low	3904	65.1	56606	67.3	
Moderate	2055	34.3	26892	32.0	
High	38	0.6	619	0.7	
Urbanization level					<0.878
Urban	1376	22.9	19166	22.8	
Suburban	2276	38.0	31781	37.8	
Rural	2345	39.1	33170	39.4	
Charlson Comorbidity Index score					<0.001
0	1262	21.0	21662	25.8	
1	2167	36.1	29135	34.6	
≥ 2	2568	42.8	33320	39.6	
Diabetes	1665	27.8	20511	24.4	<0.001
Hypertension	3640	60.7	48953	58.2	<0.001
History of alcohol intoxication	60	1.0	543	0.7	0.001
Ischemic stroke	1375	22.9	16047	19.1	<0.001
Intracranial hemorrhage	109	1.8	1218	1.5	0.022
Malignancies	298	5.0	4692	5.6	0.046
Dementia	1712	28.6	21465	25.5	<0.001

Next, we performed the multivariate Cox regression models to evaluate the adjusted HRs of death. Patients with mild TBI still had higher HR after controlling for age, gender, urbanization level, SES, diabetes, hypertension, history of alcohol intoxication, history of ischemic stroke, history of intracranial hemorrhage, malignancies, dementia, and CCI score. (1.25; 95% CI, 1.16—1.34). Other independent risk factors of death included older age, male gender, living outside of urban area, lower SES, diabetes, history of alcohol intoxication, ischemic stroke, intracranial hemorrhage, malignancies and higher CCI. The statistical results are summarized in Table 2.

**Table 2:** Adjusted HRs for patients followed from baseline to study end

**Variables**	**Hazard ratio**	**95% confidence interval**	**P-value**
Mild TBI	1.25	1.16—1.34	<0.001
Male	1.32	1.28—1.36	<0.001
Patient age	1.10	1.097—1.110	<0.001
Socioeconomic status			
Low	1	--	--
Moderate	0.30	0.28—0.31	<0.001
High	0.53	0.41—0.67	<0.001
Urbanization level			
Urban	1	--	--
Suburban	1.20	1.16—1.25	0.014
Rural	1.86	1.78—1.94	0.027
Diabetes	1.53	1.48—1.58	<0.001
Hypertension	1.00	0.97—1.04	0.962
History of alcohol intoxication	2.05	1.75—2.42	0.766
Ischemic stroke	1.29	1.25—1.34	<0.001
Intracranial hemorrhage	1.87	1.71—2.05	<0.001
Malignancies	1.98	1.89—2.08	<0.001
Dementia	0.68	0.65—0.70	<0.001
Charlson comorbidity index score			
0	1	--	--
1	1.00	0.95—1.05	0.974
≥ 2	1.46	1.40—1.53	<0.001

An analysis based on people who survived longer than 6 months was performed. There were 5113 patients in the mild TBI group and 82,262 in the control group. The HR for mild TBI was slightly decreased but still statistically significant. (1.15; 95% CI, 1.06—1.24) (Table 3).

**Table 3:** Adjusted HRs for patients who survived longer than 6 months

**Variables**	**Hazard ratio**	**95% confidence interval**	**P-value**
Mild TBI	1.15	1.06—1.24	<0.001
Male	1.34	1.29—1.38	<0.001
Patient age	1.10	1.096—1.101	<0.001
Socioeconomic status			
Low	1	--	--
Moderate	0.33	0.31—0.34	<0.001
High	0.52	0.40—0.67	<0.001
Urbanization level			
Urban	1	--	--
Suburban	1.20	1.15—1.25	0.014
Rural	1.82	1.74—1.91	0.027
Diabetes	1.53	1.48—1.59	<0.001
Hypertension	1.00	0.97—1.04	0.852
History of alcohol intoxication	1.95	1.63—2.33	0.766
Ischemic stroke	1.29	1.24—1.34	<0.001
Intracranial hemorrhage	1.85	1.68—2.05	<0.001
Malignancies	1.78	1.68—1.87	<0.001
Dementia	0.67	0.65—0.70	<0.001
Charlson comorbidity index score			
0	1	--	--
1	0.98	0.93—1.03	0.420
≥ 2	1.42	1.35—1.49	<0.001

A subgroup analysis based on patients admitted with TBI was conducted. There were 1496 patients in the TBI group and 84,117 in the control group. After controlling for the same covariates, the HR of death in the hospitalized group was higher than for those discharged directly after visits. (HR 1.70; 95% CI, 1.52—1.91). The statistical results of other covariates were similar to those with the primary study cohort and are summarized in Table 4.

**Table 4:** Adjusted HRs for patients admitted with TBI

**Variables**	**Hazard ratio**	**95% confidence interval**	**P-value**
Mild TBI	1.70	1.52—1.91	<0.001
Male	1.32	1.28—1.36	<0.001
Patient age	1.097	1.095—1.100	<0.001
Socioeconomic status			
Low	1	--	--
Moderate	0.29	0.28—0.30	<0.001
High	0.55	0.43—0.70	<0.001
Urbanization level			
Urban	1	--	--
Suburban	1.20	1.16—1.26	0.014
Rural	1.86	1.78—1.94	0.027
Diabetes	1.55	1.49—1.60	<0.001
Hypertension	1.00	0.97—1.04	0.951
History of alcohol intoxication	2.05	1.73—2.42	0.766
Ischemic stroke	1.30	1.26—1.35	<0.001
Intracranial hemorrhage	1.91	1.74—2.09	<0.001
Malignancies	1.99	1.90—2.10	<0.001
Dementia	0.67	0.65—0.70	<0.001
Charlson comorbidity index score			
0	1	--	--
1	1.00	0.95—1.05	0.420
≥ 2	1.46	1.39—1.53	<0.001

**Figure 3:**
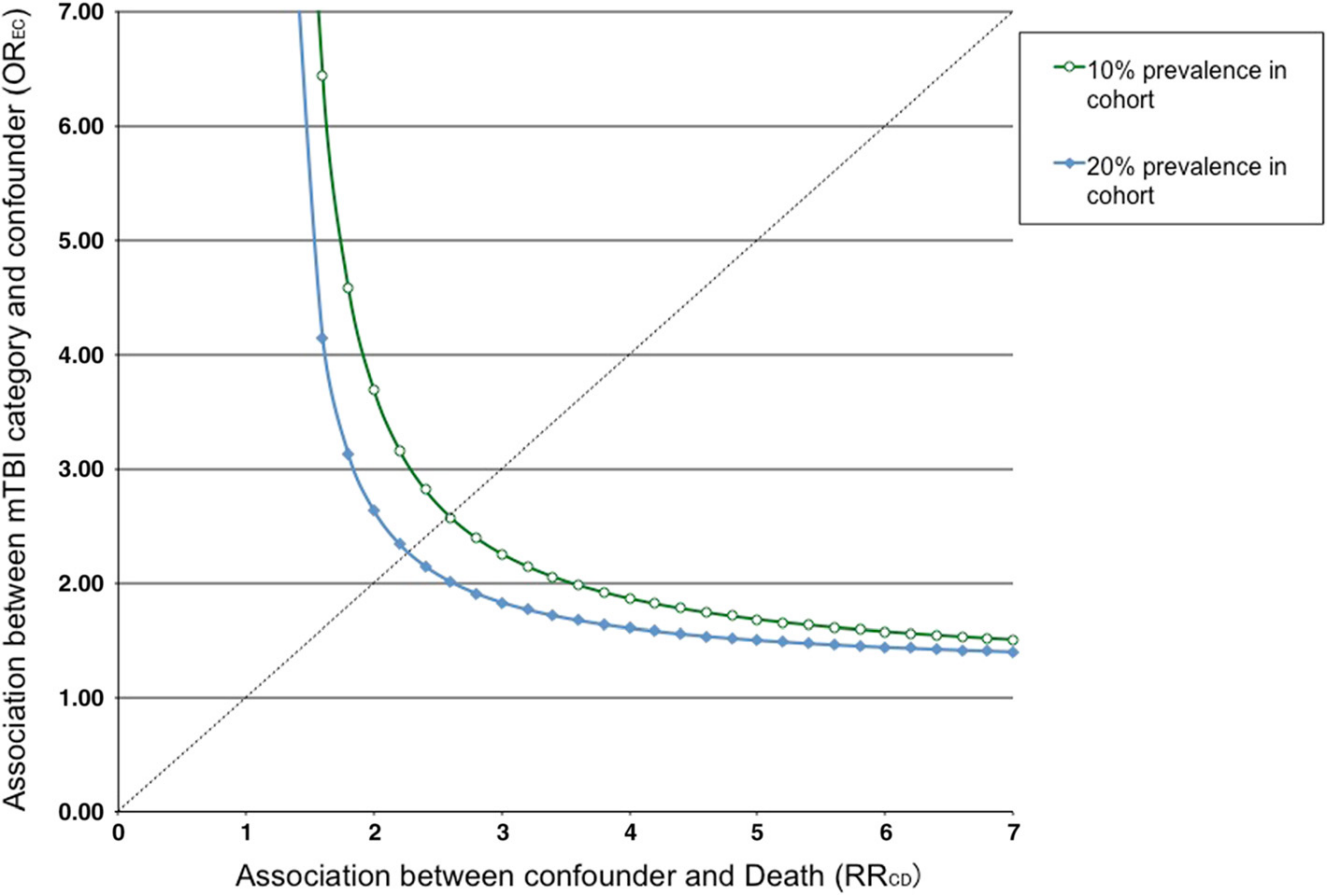
Sensitivity analyses of an unmeasured confounder.

Sensitivity analyses showed that an unmeasured confounder present in 10% of the study population would be required to elevate the risk of death by a factor of 2.6 and would also have to have a prevalence among patients with mild TBI that would be around 2.6 times that among the control group to explain a lower 95% confidence limit HR of 1.16 (Figure 3).

## Discussion

Studies have found that TBI is associated with increased risk of death especially in the elderly [3,4,7,9,19]. Although the actual mechanism is still not completely understood, cerebrovascular atherosclerosis during aging could increase the risk of injury and induce a secondary insult. Moreover, decreased free radical clearance in the elderly may further increase oxidative damage after TBI [20,21]. McIntyre et al., [4] performed a meta-analysis that included 24 studies and found that moderate to severe TBI had higher odds ratios for death than mild TBI in the elderly. In a study by Brown et al., [9] the HRs for death in patients with moderate to severe TBI were significantly higher, but the study failed to determine whether mild TBI had higher HRs. In our study, we further extended the impact of TBI to the mild type, utilizing the largest cohort study to date and found that patients with a single mild TBI have a higher risk of death later in their lives compared to the general population. Our study had enough statistical power to provide a precise estimate of the HR (1.25; 95% CI, 1.16—1.34), which was statistically and clinically significant. The database corresponds well to the whole population; therefore, loss of follow-up or selection bias were not concerns.

Another advantage of this study is that we directly compared mild TBI patients with the general population simultaneously by survival analysis. Using this design, we were able to adjust extensively for possible confounding factors. Among the covariates, older age, male gender, lower SES, diabetes, stroke and malignancies were found to be associated with higher mortality rates in older patients with TBI, which is consistent with previous publications [3,5,22]. Of note, hypertension and dementia were not associated with increased risk of death in our study. The possible reason is that their effects on death may be partly explained by coexisting comorbidities or the CCI score [13].

Several limitations were associated with this study. First, our findings were derived from administrative data. Cases were collected using ICD-9-CM diagnosis codes, which is good for insurance reimbursement but not a substitute for precise operative definition. Therefore, the validity of the diagnosis (i.e., sensitivity, specificity and accuracy) was not fully assessed. In Bazarian et al. [12] the sensitivity of ICD-9-CM codes for mild TBI was 45.9% with a specificity of 97.8%. In other words, people in the mild TBI group were highly likely to have mild TBI, while some individuals in the control group may have had mild TBI during the study period but the inclusion strategy failed to identify them. In this situation, the predicted effect of mild TBI on death should be toward the null, but we still found a significant risk of death in patients with mild TBI. Second, we were not able to obtain the clinical information for patients with mild TBI, such as the Glasgow Coma Scale score, findings on cranial CT, the injury mechanism and the initial presentations. By definition, labeling our cases as ‘mild TBI’ may have been inappropriate. However, it has been validated that the ICD9-CM codes have high specificity regarding diagnosis of mild TBI [12]. Furthermore, we excluded those patients who were admitted to the hospitals to make sure that the patients enrolled were really in the “mild” category. Based on our inclusion criteria, although not totally precise, we think the cases in our study group were highly correlated with the definition of mild TBI [13]. Also, although we extensively adjusted for possible comorbidities, unmeasured cofounding is still an issue. Based on the sensitivity analyses, the adjusted HR was significant enough that the effect of residual confounding should be stronger than any of the covariates we included in order to avert the estimated risk. Furthermore, we did a subgroup analysis and found a higher HR of death (1.70) in patients admitted with TBI and the unmeasured confounding could not fully explain the ‘dose-response’ of injury severity.

## Conclusions

Mild TBI is an independent significant risk factor of death in the elderly. The result indicates that more emphasis on head injury prevention would be worthwhile.

## Abbreviations

TBI: Traumatic brain injury; HR: Hazard ratio; NHI: National Health Insurance; SES: Socioeconomic status; CCI: Charlson Comorbidity Index.

## Competing interests

The authors declare that they have no competing interests.

## Authors’ contributions

P-L C study concept and design. H-Y L manuscript formation. Y-K L study supervision. C-Y H data analysis. C-C L data analysis and study supervision. Y-C S analysis and interpretation. All authors read and approved the final manuscript.
